# Forecasting distributed energy resources adoption for power systems

**DOI:** 10.1016/j.isci.2022.104381

**Published:** 2022-05-10

**Authors:** Nicholas Willems, Ashok Sekar, Benjamin Sigrin, Varun Rai

**Affiliations:** 1 National Renewable Energy Laboratory, 15013 Denver W Pkwy., Golden, CO 80401, USA; 2 Walker Department of Mechanical Engineering, The University of Texas at Austin, 204 E. Dean Keeton St., Austin, TX 78712, USA; 3 LBJ School of Public Affairs, The University of Texas at Austin, 2300 Red River St E-2700, Austin, TX 78712, USA

**Keywords:** Energy resources, Energy policy, Energy management, Energy Modeling, Energy Systems

## Abstract

Failing to incorporate accurate distributed energy resource penetration forecasts into long-term resource and transmission planning can lead to cost inefficiencies at best and system failures at worst. We have developed an open-source tool that employs an advanced Bass specification to calibrate and forecast technology adoption. The advanced specification includes geographic clustering, exogenously estimated market size, and dynamic time steps. Training on historical adoption of rooftop photovoltaics at the U.S. county-level and using detailed techno-economic estimates, our model achieves a two-year average mean-absolute-percentage-error of 19% in predicting system counts at the county-level, weighted by population. Model error was negatively correlated with market maturity—the error was 12% for counties in states with at least 28 W-per-capita of installed capacity. The advanced specification significantly reduces unweighted forecasting percent error compared to a conventional Bass specification: from 196% to 25% for capacity and from 226% to 22% for system count.

## Highlights

• Developed open-sourced tool to calibrate and validate forecasts of residential PV
• The method introduces three improvements to the traditional Bass model
• Demonstrate improvement to accuracy by > 170% compared to traditional specification
• Model achieves mean absolute percentage error of <20% for system counts

## Graphical abstract

**Figure:**
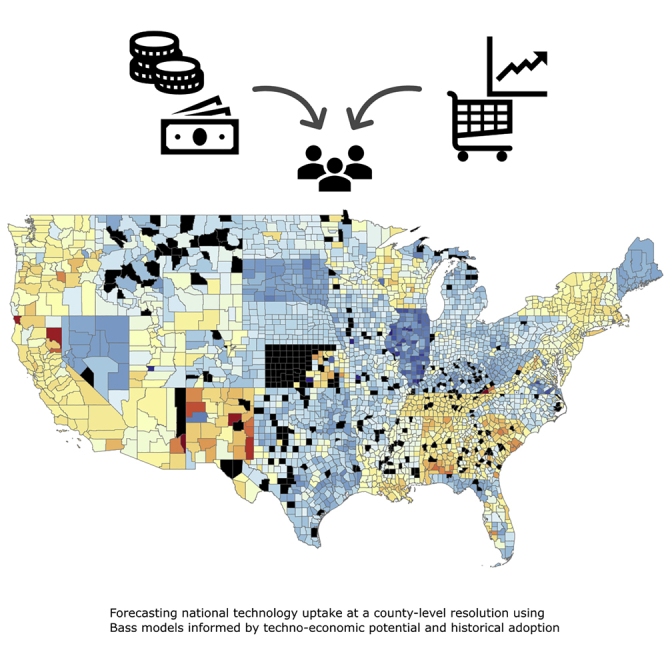

## Introduction

Distributed energy resources (DERs) such as solar, wind, and batteries are one of the largest sources of new electricity capacity in the United States, and this trend is expected to continue in the future (EIA, 2021a; International Energy Agency, 2020). Because DERs can also help achieve decarbonization and climate goals, regulators (e.g., public utility commissions [PUCs]) increasingly require incorporation of DER adoption forecasts into power system (i.e., distribution and integrated resource) planning (Jenkins et al., 2018; Kittner et al., 2021; Williams et al., 2012). Forecasting DER penetration, in total number of units installed and system size, is important for several reasons. First, identifying areas where the technology will be adopted informs utilities’ investments in other energy-system infrastructure such as transmission and distribution infrastructure. Second, quantifying the net change in electricity consumption offset by DER generation will serve to better understand supply requirements and thus to manage associated financial risks. Finally, forecasting DERs is key to optimally integrating these resources into the grid. This includes helping develop a more accurate spatiotemporal picture of demand which, in turn, is expected to be central to maintaining system functionality generally and especially during extreme grid conditions (Hanser and Van Horn, 2016, p. 20; Horowitz et al., 2019).

Both under- and over-forecasting DER penetration can have consequences for power system planning, especially in areas where there is significant uptake of DERs. Over-forecasting can lead to insufficiently provisioning alternative bulk generation and result in a less dependable system. Under-forecasting may result in the opposite problem: spending more than is necessary by building superfluous generation and infrastructure. Forecasts with poor spatial fidelity of where DERs will be adopted can hinder specific circuits receiving necessary distribution upgrades (Horowitz et al., 2019). For these reasons, to aid systems planning, it is essential to develop sophisticated DER adoption models with the flexibility to quantify both capacity and spatial distribution under several real-world scenarios. In bulk power system planning alone, low-quality DER-adoption forecasting could cost up to $7/MWh of electricity sales (Gagnon et al., 2018), which is about 21% of average wholesale generation price in the U.S. for the year 2020 (EIA, 2021b). That is, for a single large utility with moderate growth in DER penetration, costs could be on the order of $30 million over a 15-year period (Gagnon et al., 2018).

This paper presents a new method to calibrate technology adoption forecasting models using a Bass specification. In particular, we use historical residential solar photovoltaic (PV) adoption data by county-sector alongside detailed estimates of technical and economic potential to forecast nationwide residential rooftop solar adoption at a fine spatial scale. The method produces estimates of both system counts and capacity by evaluating the attractiveness for representative individuals. This method is implemented in the Distributed Generation Market Demand Model (dGen), an open-sourced tool developed by the National Renewable Energy Laboratory (NREL) (NREL, 2016). The tool is in active development and is used by several organizations for power systems planning. Specifically, dGen is used for forecasting rooftop PV, storage, and/or distributed wind adoption across countries including the United States (U.S.), Mexico, and India. Here, we focus on results around forecasting residential solar PV in the U.S.

Given the significance of forecasting and the widespread use of the dGen tool, the broad focus of this paper is to demonstrate the capability of the new underlying calibration and forecasting method and situate it within existing body of DER adoption literature. To that end, next we present a brief background on models used to predict DER adoption, with specific focus on Bass diffusion. Then, we give an overview of the composite data used to calibrate and validate the model (Data). In Modeling, we detail the calibration algorithm itself, describing how it is used for forecasting, and how those forecasts are validated. We specifically focus on how the calibration and forecasting approach differs from a traditional Bass diffusion implementation. Finally, we close with forecasting results and discussion.

### Predicting distributed energy resource adoption

Existing DER adoption forecasting efforts can be studied using three different lenses. The first lens considers the approach used for building the model, i.e., a top-down approach or a bottom-up approach. A top-down approach uses macro-level indicators to model market forecasts, whereas a bottom-up approach uses micro-level indicators to model individual forecasts, which are then aggregated into a market forecast. The difference between macro-level and micro-level indicators is their specificity: micro-level indicators represent the traits of a fairly granular unit—typically an individual or a household, but it can also be a small spatial area such as a block—within the market, and macro-level indicators represent an aggregate market, such as a county, state, or even an entire country, to begin with. The fundamental consequence of this difference is that a top-down approach cannot model individual-level adoption, whereas a bottom-up approach can. However, micro-level indicators are more difficult to collect and process at scale, making top-down models easier to execute. Three classes of top-down models are popularly used to forecast DER deployment (Horowitz et al., 2019)—econometric, time series, and Bass diffusion. Econometric models use observed relationships between dependent and independent variables, either for explanation or prediction, e.g (Bernards et al., 2018; Davidson et al., 2014; Dharshing, 2017). Time-series models extrapolate from historical data to infer future outcomes (Dong et al., 2017). They are the simplest specification to use because they only require past observations, though typically are only useful in near-term forecasting. Lastly, Bass models are among the most widely used specifications because they are simple to parameterize and are intended to simulate diffusion of new technologies. Meanwhile, agent-based models (Rai and Henry, 2016; Rai and Robinson, 2015; Sigrin et al., 2016; Zhang et al., 2016) are the most common bottom-up approach for forecasting DERs.

The second lens for understanding extant DER adoption forecast methods examines the model specification: the relationship between the indicators and the outcome. Broadly, there are two specifications, theory-driven and data-driven. Theory-driven models impose a relationship between the indicators and the outcome based on a theory of individual or market behavior. Data-driven models, on the other hand, are agnostic and ideally expose hidden relationships in the data that explain outcomes better than theory. This is the foundation of machine learning, which has demonstrated superior predictive accuracy compared with theory-driven approaches (Horowitz et al., 2019). However, data-driven models have several drawbacks including: requiring large amounts of data, susceptibility to overfitting, and decreased interpretability. In contrast, theory-driven models are diagnostic in nature and can help decision makers understand the drivers and barriers of DER adoption/non-adoption while also serving as a tool for evaluating the impact of different policy interventions. Because the forecasting of DER adoption typically involves projecting decades into the future, it is difficult to assess superiority of a specification because model validation can only be applied retrospectively (Yu et al., 2018; Zhang et al., 2016).

The third lens through which to evaluate DER adoption forecasting models assesses their capability. While the primary goal is forecasting adoption, it is also important that models are adaptable, scalable, and have sufficient spatial resolution, all while remaining sensitive to changing policy contexts, incentives, and techno-economic conditions. For example, Rai and Robinson (2015) present a highly granular agent-based model of residential solar PV adoption at the scale of a utility service territory (Austin, Texas). Their model incorporates not only economic but also physical and social household-level determinants of residential PV adoption. While the model is calibrated and validated across multiple outcomes, computational cost and data requirements make this model difficult to scale and adapt to different geographies. Using a different approach, Yu et al. (2018) generate a predictive model of solar adoption based on myriad socioeconomic features. The model covers the entire contiguous United States and provides results down to the census tract level, but it is trained on cross-sectional data and not used for forecasting future adoption. On the other hand, Williams et al. (2020) model annual PV installations as a function of net present value for five different international regions (three U.S. states and two countries). Given regional economics, the model is adaptable and highly scalable; however, spatial resolution of the model is quite coarse.

### Bass diffusion

The Bass diffusion model describes first purchases (i.e., adoption) of a new, durable technology. It is a top-down, theory-driven modeling approach and the specific implementation we present here (3) is both adaptable and scalable. It has been used to forecast adoption of not only consumer durables but also, among many others, telecom equipment, semiconductor chips, and medical products (Bass, 2004). The premise of the model is that the likelihood that new customers will adopt a technology (LHS of Equation 1) is a function of the cumulative number of past adopters (RHS of Equation 1), where *F* is the fraction of market adoption (i.e., installed base fraction) compared to ultimate size of the market (*m*). Bass posited that there are two types of adopters: innovators and imitators. Innovators are adopters in the early phases of a technology’s diffusion process who act independently of other individuals. Imitators, on the other hand, are assumed to adopt the innovation under social influence from extant adopters of the innovation. Initially, innovators are the only adopters, but as the number of past adopters increases, so too does the probability of new imitator adoption. Thus, innovators exert the most influence when the product is new; over time, their influence fades as more imitators purchase the product. Total adoption is limited by the size of the market which the model approaches asymptotically (Bass, 1969).

(Equation 1) $$\frac{\frac{dF\left(t\right)}{dt}}{1-F\left(t\right)}=p+qF\left(t\right);\;F\left(0\right)=0$$

The solution to Equation 1 is the Bass model, which poses *F* as a function of two parameters: the coefficients of innovation (*p*) and imitation (*q*) and one variable: time (*t*) (Equation 2). The product of installed base fraction and market size gives the total number of adopters (Equation 3). The coefficients of imitation and innovation reflect the relative mixture of internal and external influences on adoption, where *p* corresponds to the probability that a technology innovator will adopt independently of other adopters and *q* the probability that an imitator will adopt, proportional to the existing base of diffusion (*F*). The relative magnitudes of *p* and *q* influence the rate of saturation and shape of diffusion over time. In a basic Bass model, these parameters are static. This is in conflict with real-world technology adoption where more customers are attracted as the technology improves and consumer expectations may be time-varying (Zhang et al., 2020). Under typical *p* and *q* parameter values, the diffusion curve results in a classic symmetrical sigmoid “S-curve.” The market size parameter linearly scales the diffusion curve, corresponding to the technology’s total addressable market.

(Equation 2) $$Installed\;Base\;Fraction,\;F\left(t\right)=\frac{1-e^{-\left(p+q\right)t}}{1+\frac{q}{p}e^{-\left(p+q\right)t}}$$

(Equation 3) Totalnumberofadoptersattime(t)=F(t)∗m

Bass parameters may be estimated by regression on historic sales data over time, though market size is commonly estimated exogenously. The objection to estimating market size endogenously is based on the difficulty of using only a few observations of sales for estimating the total addressable market, which might happen years or even decades after the initial introduction of the innovation. Thornier still are issues of technology evolution—for example, later technology versions may be secularly superior to earlier versions, or a technology’s relative advantage could depend on the broader market context (e.g., policy interventions and competing innovations), which itself could change significantly over time. In the context of DERs, the exogenous estimate of market size is typically formulated in three different ways: the total building stock; a technology’s technical potential (Kurdgelashvili et al., 2019); or, most commonly, as a function of the economic attractiveness (Dong and Sigrin, 2019).

#### Application and extensions

The power and critique of the Bass model both lie in its parsimony (Bass, 2004). According to an analysis of 213 applications conducted by Sultan et al. (1990), a meta-analytic Bass model can explain “30% to 50% of the variability in estimates of the parameters in the diffusion model” suggesting the power of the model. A more recent example—wherein automobile sales were forecasted using a Bass model—showed predictive accuracy of about 97% (Zhang et al., 2020). In contrast, the generalized Bass model (GBM) incorporates additional exogenous influences on technology adoption, commonly marketing mix and product price (Bass et al., 1994). In theory, GBMs can include an arbitrary number of covariates, for instance, time-based effects on price as modeled in the market size parameter.

In specifying the Bass model, practitioners face subjective decisions about the degree of market segmentation based on socio-economic, cultural, and behavioral aspects (Peres et al., 2010; Van den Bulte and Stremersch, 2004). For instance, Kurdgelashvili et al. (2019) found significant differences in the Bass parameters by county in California and it is intuitive to expect that markets will differ in the rate of diffusion based on the population composition. Conversely, fitting the Bass model on adoption within larger populations can help to smooth out underlying randomness in small-population diffusion processes (Peres et al., 2010). Another critique of the Bass model is its poor ability to simulate step changes in market conditions, for instance, introduction of a new policy to incentivize adoption. The classic Bass model—with an endogenous market size estimate—has no capability to reflect this step change. Researchers have addressed the issue of changing market potential by quantifying it in several ways, including exogenously as a function of observed variables (e.g., price, or as a monotonic/stepwise function of time), and dynamically, by considering both exogenous and endogenous variables. Velickovic et al. (2016) provide a detailed literature review on this topic. In addition, exogenous shocks can impact diffusion (e.g., technological breakthrough that significantly reduces technology cost). In these cases, it is important to “reset” the diffusion curve to reflect that the technology is now diffusing into a new total addressable market.

These factors suggest that a better fit could be achieved through three adaptations to the traditional Bass specification: segmenting the market to capture heterogeneity, calculating market potential exogenously by considering policy and economic outlook, and treating time dynamically by considering the “equivalent” amount of time that diffusion has occurred. Together, the latter two adaptations capture market conditions. As noted above, previous studies have devised more performant Bass models by using market segmentation (e.g., Kurdgelashvili et al. (2019)) and dynamically calculated exogenous market size (see Velickovic et al. (2016) or Zhang et al. (2020)); we are not aware of any previous work which used a dynamic time variable. To our knowledge, these three improvements have not yet been combined and tested by other researchers for solar PV or any other technology. Later (Validation), we provide a comparison of the method presented here to related literature.

## Data

### Ground truth historical adoption

We combine three sources of historical solar adoption data to build out the ground truth dataset, which describes cumulative and yearly adoption for all counties in the contiguous United States from 2010 through 2020. The central dataset comes from the *Tracking the Sun* (TTS) report published biannually by Lawrence Berkeley National Laboratory (Barbose et al., 2019, 2020). This dataset provides system-level details on installations dating back to the 1990s for much of the country. TTS is not totally comprehensive, however, so we supplement it with estimates from the Wood Mackenzie and Solar Energy Industries Association *U.S. Solar Market Insight* (USSMI) (U.S. Solar Market Insight, 2020) report, as well as the DeepSolar Project (Yu et al., 2018). The USSMI provides quarterly estimates of capacity and installations for most states. Meanwhile, DeepSolar used satellite imagery and image recognition to identify the locations of solar panels across the country in 2017. While some of these sources include Alaska and Hawaii, for the purposes of our modeling, we focus on the contiguous United States.

The key challenge in assembling the necessary ground truth data was in successfully matching county-level resolution at national scale over time. Namely, we required a county-level resolution, at a national scale, for all years between 2014 and 2020—something that none of the three datasets could offer alone. With TTS nearest to meeting all three criteria, we use it as a starting point. Because TTS data are geolocated by city and/or ZIP code, we use national crosswalks (available from the U.S. Census Bureau and Housing and Urban Development/U.S. Postal Service, respectively) to match these identifiers to counties. About 88% of the data have valid system and location information and can be mapped uniquely to a county. A remaining 6% lie in ZIP codes that span multiple counties—these are probabilistically assigned to one county based on the proportion of all the addresses in the relevant counties. After cross-checking for inadmissible results (i.e., county-state combinations that do not exist), we are left with 94% of the original data points mapped to counties.

To address the underlying incompleteness of the TTS data, we compare the state-level totals to the USSMI dataset. In states where TTS records a higher count, we maintain the granularity therein and treat the data as complete; in states where TTS records a lower count, the additional values from USSMI are imputed across the state’s member counties. We follow an imputation scheme which aims to reduce two types of documented error in TTS: completely missing incentive programs and cessation of reporting by existing programs after 2017. To address the first type of error, we impute up to 2017 using DeepSolar (2017 vintage). Namely, we calculate the (county) difference in system counts between DeepSolar and TTS (2017) and, where positive, use it as a weighting factor for imputation. As TTS collects data from specific programs, this approach aims to fill the gaps where program data were never reported. Because DeepSolar provides a snapshot (i.e., cumulative installations in 2017), it captures only counties which were missing data in 2017. Over time, the coverage of TTS has decreased: in 2018, only 25 states reported any incremental data (Barbose et al., 2019, pp. 7, 39–41). The implication of such sparse reporting is that, in a given state, there are likely *more* counties with missing installation data with each passing year. Using the DeepSolar weighting in subsequent years ignores this fact by apportioning installations only to counties which were missing in 2017. Instead, in the absence of another (DeepSolar-like) snapshot of adoption at 2020, we use population as a weighting factor after 2017. In particular, we consider all counties with a nonzero number of (previously imputed) installations and impute the additional values from USSMI according to their relative population. The rate of imputation increases over time, with just 13 states imputed in 2010, 30 in 2015, and 46 in 2020; the number of added systems is significant: median 30% of TTS total (this is not surprising given e.g. (Barbose et al., 2019, p. 7)).

**Figure 1:**
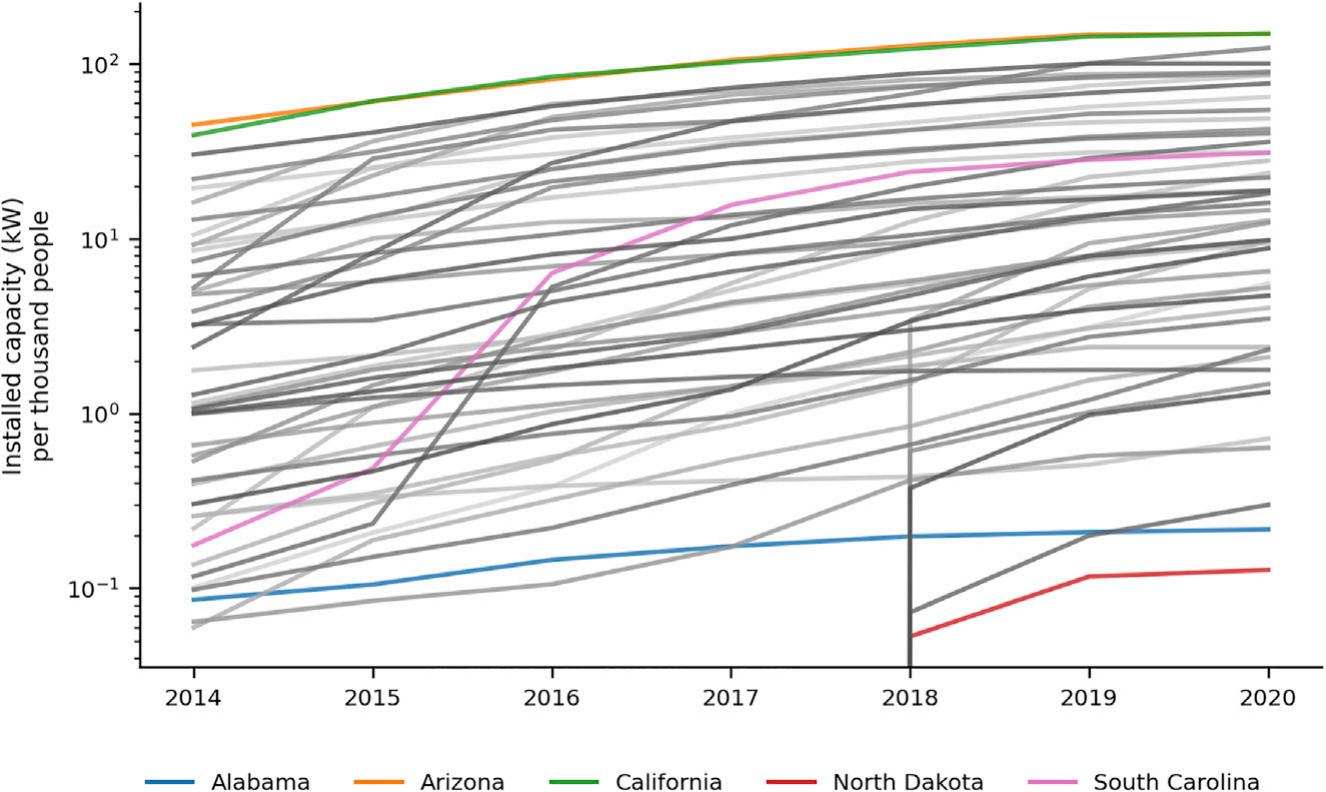
State profiles of historical distributed PV adoption Statewise historical adoption of distributed solar PV from 2014 to 2020. A handful of states are shown in color to highlight the large variation among states in the temporal profile of adoption.

By following this methodology of building on the information present in TTS by incorporating two other primary sources, we generate a dataset of historical (2010–2020) solar adoption for all counties in the continental United States. Both the scale and rates of adoption vary widely across the country (Figure 1), spanning three orders of magnitude. By 2020, California and Arizona (green and orange) reach more than 149 kW per thousand people, while North Dakota and Alabama (red and blue) remain under 0.3 kW per thousand people. A good illustration of the significant variation in rate of adoption across states is the contrast between Alabama and South Carolina, both of which start at approximately 0.1 kW per thousand people in 2014; whereas Alabama (blue) has only modest additional growth by 2020, South Carolina (purple) has nearly 15 kW per thousand people by 2020. This wide variation in both total magnitude and rate of change presents a challenge for model formulation and calibration.

### Data limitations

In the dGen model, residential solar only encompasses adoption by single-family homes. This is an inherent point of conflict between dGen and data sources (TTS, USSMI, and DeepSolar), where “residential solar” could include larger systems designed for multifamily buildings. To account for this difference, we prefilter the TTS data and correspondingly scale the USSMI data. Specifically, starting with installations labeled “residential”, we remove TTS installation records in the 95^th^ percentile of size (>∼13 kW), which comprise about 22% of total capacity reported in TTS. To match this, before imputation (see Ground truth historical adoption above), we also scale the state number of adopters and capacity totals of USSMI by 0.95 and 0.78, respectively.

An additional challenge in making the data suitable for use in dGen is translating county adoption to agent-level adoption. In dGen, the term agent is not used in the sense of “agent-based” modeling but rather refers to a decision-making unit. In theory, the unit can represent an individual consumer or a statistically representative aggregation of individual consumers. Owing to data limitations at the individual-level, a dGen agent currently represents an aggregate population at the sub-county level. The agent development process involves the use of a statistical framework to aggregate individuals into agents and assign attributes (such as available roof area, annual energy consumption, and electricity price) that are necessary to make the investment decisions. A detailed description of the agent development process is provided in Sigrin et al. (2016). County-level historical adoption (ground truth data) is divided among that county’s constituent agents based on their available roof area which accounts for the number and type of residences (e.g., single family homes) (Gagnon et al., 2016; Sigrin and Mooney, 2018).

Other shortcomings in the primary data themselves and our own methodological assumptions potentially contribute to additional sources of error in these data. First, although we use TTS as the foundational dataset, there is no guarantee it—or the other two datasets—is free from omissions, misprints, or duplicates. Most prominently, TTS does not actively collect data on solar deployment but instead relies on self-reporting from state and local solar programs. Second, when identifying TTS installation counties by zip-code, we use a recent crosswalk for all years, but zip-code designations themselves change over time. Because these changes are local in nature, we expect errors of this type to be small. Third, we are aware that the DeepSolar project only examined census tracts with a nighttime light intensity (a proxy for population density) above a minimum threshold (Yu et al., 2018, p. 2607) and therefore does not strictly cover the entire area of the United States. We limit the effects of this by using data from Yu et al. (2018) only to develop a weighting factor. Fourth, in imputing additional values from USSMI to the TTS data after 2017, we use population as the only predictive variable. The objective of imputation is to account for the discrepancy between the TTS and USSMI state totals. While drivers of adoption are well documented (e.g., population density, income (Yu et al., 2018)), the drivers of this error are not known. In weighting the excess solar count by population (as opposed to, for example, income), we assume that larger counties (rather than wealthier) exhibit a larger discrepancy. The effect of this weighting factor is that the relative difference in the county-level solar installations per-capita is unchanged, which is one way to preserve the underlying structure of the adoption patterns observed in the data.

## Modeling

Our central contribution to the literature is developing an open-source method to calibrate and validate the predictive model of customer adoption implemented in the dGen model. This leverages many of the dGen outputs and functionality described in the introduction and the model description (NREL, 2016; Sigrin et al., 2016), as well as incorporating the new data sources outlined in Data. The calibration algorithm proceeds in three steps: grouping, tuning, and disaggregating. First, the agents are grouped and aggregated to form larger representative markets. Next, the parameters of a Bass diffusion curve are tuned for each group based on previous (first historical and later forecasted) adoption. Lastly, the group-level Bass model is used to predict agent-level adoption. The calibrated model is used to forecast future adoption, and outputs are validated against unseen historical data from 2019 to 2020. This strategy of grouping, tuning, and disaggregating—our key contribution—is generalizable to calibrating Bass models of any other technology. Although implemented in NREL’s dGen model, the calibration method depends on dGen only for exogenously estimating market size (Market size).

### Calibration

A typical Bass diffusion model uses a single set of calibrated coefficients of innovation (*p*) and imitation (*q*) and market size (*m*), and advances linearly through time (Bass, 1969). The calibration method presented here deviates from the traditional approach in three ways (Table 1). First, to achieve better resolution of Bass parameters (*p* and *q*), we calibrate these values for distinct groups of agents (Bass parameters). Second, rather than calibrating market size alongside the Bass parameters, market size is determined exogenously based on economics (Market size). Lastly, we use a so-called “equivalent time” parameter that allows time to advance nonlinearly and helps account for changes in market conditions (Time). This approach differs from producing a Bass model for each state in two important ways: first, the market size and equivalent time are determined for each individual agent; second, prediction is still done at the agent—as opposed to group—level.

**Table 1:** Comparison of the modeling adaptation to a traditional Bass modeling approach

	Traditional Bass Model	Adaptations
Calibration Level	National	State
Market Size	Calibrated	Exogenously modeled (informed by other dGen model components)
Time	Linear	Equivalent

#### Bass parameters

Considering dGen is a national-scale model, rather than applying the same Bass parameters everywhere, one may allow *p* and *q* to vary across distinct markets within the nation. The grouping module permits markets defined by sets of arbitrary geographic scales and non-geographic attributes. For interpretability, we use U.S. states to define distinct markets and capture heterogeneity in patterns of solar adoption across the country. There are several benefits of grouping agents into state-level markets. First, because Bass diffusion describes a bulk phenomenon, there is no reason to expect individual agents (who represent small numbers of individuals) to follow a smooth diffusion curve; aggregating agents into larger groups is more consistent with the underlying theory, and Bass diffusion patterns are more likely to be observed. Second, optimizing parameters for large groups reduces computational expense. Thirdly, U.S. states tend to have some degree of within-state homogeneity due to similar policies/incentives within states. Finally, no significant improvement in results was found when agents were clustered into groups based on non-geographic characteristics (e.g., payback period, price of electricity, wealth, and percent of buildings developable). That being said, should modelers expect homogeneity across other (possibly non-geographic) market groupings, the approach presented here is flexible to arbitrary clusters.

##### Parameter tuning

Because Equation 2 assumes diffusion begins at time zero, it is necessary to estimate an additional parameter, *t*_*0*_ (the time offset, where *t* = *t*_*i*_ + *t*_*0*_), to calibrate values of *p* and *q*. While this is possible given several values of installed base fraction *F* at various times *t*_*i*_, it is computationally more tractable to estimate fewer parameters. An alternative approach eliminates one of these free parameters (*t*_*0*_) by using a discretized approximation of the differential form of the Bass model. This approximation is derived by starting with the basic Bass formulation (Equation 1) and replacing the derivative with its forward difference approximation:

(Equation 4) $$F\left(t+\Delta t\right)\approx F\left(t\right)-\Delta t\left(qF{\left(t\right)}^{2}+\left(p-q\right)F\left(t\right)-p\right)\;$$

By approximating the derivative, this form of the Bass model requires knowledge only of the distance between time steps (Δ*t*) but not the time offset (*t*_*0*_) itself. The installed base fraction, F(t), is bounded on either side by 0 and the maximum market share. Given Equation 4, it is possible to conduct a simple optimization of *p* and *q* in [F(t),F(t+Δt)] space. Given the known sensitivity of estimating Bass model parameters from historic data, we enforce constraints on the parameter estimates. Namely, we restrict the parameters *p* ε [0.0001, 0.002] and *q* ε [0.2, 0.4] to ensure the resulting diffusion patterns are reasonable. These bounds imply that peak adoption occurs [13.7, 38.0] years after the first year of diffusion. The parameter tuning module accepts as input the agents with their assignment to a group. These agents are aggregated, yielding a group-level yearly adoption fraction, which is used to calibrate Bass parameters via the simplified, discrete differential form given above. All agents in a group share the same Bass parameters.

#### Market size

Rather than calibrating market size alongside the coefficients of innovation (*p*) and imitation (*q*), a value is derived from other components of the larger dGen model. Specifically, each agent’s payback period is estimated based on models of the local economics of solar as described in the study by Sigrin et al. (2016). The model uses technical potential estimates based on the combination of single-family building stock and lidar-based estimates of rooftop suitability for solar (Gagnon et al., 2016). The solar economics are calculated in the PySAM model (Gilman et al., 2019) using hourly synthetic load and solar generation profiles for each agent (Sigrin et al., 2016), the agent’s retail rate structure (Open Energy Information, 2020), applicable existing incentives (NC Clean Energy Technology Center, 2019), and technology cost and financing data (NREL, 2019). Then, payback period is mapped to the market size parameter (*m*), a.k.a. “maximum market size” or “total addressable market,” based on empirical customer willingness-to-pay surveys (Dong and Sigrin, 2019). The exogenously specified market size provides an upper bound for the Bass parameter calibration (Parameter tuning) and influences the equivalent time parameter (Time).

Using an exogenously specified market size has some advantages compared to calibrating based on historical adoption. First, it is a natural way to represent the underlying heterogeneity in the economic circumstances of the agents. Second, models of these circumstances can include information about the future—such as expiring incentives or programs running out of money—rather than assuming future conditions mirror the past. Including such detailed nationwide agent economics is also a novel contribution of this paper: for example, Yu et al. (2018) use only the number of years, since each incentive began as an indicator, whereas Williams et al. (2020) characterize national incentives for Japan and Germany and state incentives for Arizona, California, and Massachusetts. Finally, as with using the differential Bass formulation, this approach avoids computational issues with optimizing many parameters at once. It should be noted that future market size calibration does require assumptions about future economic conditions, for which we use the Energy Information Administration’s Annual energy outlook and the Annual Technology Baseline low-cost PV scenario (Annual Energy Outlook, 2018; NREL, 2019).

#### Time

A step beyond encoding information about dynamic market conditions in the market size is allowing the model to proceed in dynamic time steps. In a traditional Bass model, the market size is a fixed quantity. Allowing market size to evolve over time—with changes in demographics and policy—calls for a time variable which takes these dynamics into account. This added complexity reduces error in validation and gives more intuitive long-term forecasts. We derive the appropriate time variable by considering the classical solution to the Bass model—in terms of installed base fraction *F* (Equation 2)—and recognizing that *F* can be expressed as the ratio of the number of adopters and market size. Solving for t yields what we term the “equivalent time”, or the equivalent point on the diffusion curve given the current market conditions (Equation 5). The equivalent time captures variation in the total market potential as policy and economics change. Importantly, this variable is a function of the current market conditions and calibrated Bass parameters—it does not constitute an additional free parameter in calibration.

(Equation 5) $$t_{equiv}=\frac{-1}{p+q}\ln\left(\frac{1-\frac{number\;of\;adopters}{market\;size}}{1+\frac{q}{p}\cdot \frac{number\;of\;adopters}{market\;size}}\right)\;$$

To understand how equivalent time allows the model to advance nonlinearly, consider a scenario where a new policy (e.g., increased rebates) is introduced to a mostly saturated market. A linear time variable would indicate that the market was already saturated and therefore little to no diffusion would occur in subsequent timesteps. To address this shortcoming, equivalent time is responsive to the change in market size and effectively resets the model to the equivalent point on the diffusion curve given the new market conditions. We assume all adoption is sunk, so there is no corresponding scenario where agents un-adopt.

### Forecasting

Given Bass parameters calibrated at the state level (Bass parameters), exogenously specified market size for each representative agent (Market size), and an equivalent time for each representative agent (Time), adoption is forecast in 2-year increments. Each agent represents a collection of like individuals, so, in this context, adoption is considered as a continuous variable or as the fraction of the agent’s weighting factor. Likewise, the market size represents the number of potential adopters among a type of individual in a county. The amount of solar adoption by a particular agent in the next time step is given by the Bass model employing the calibrated parameters (Equation 6):

(Equation 6) F(t+2)=1−e−(p+q)(tequiv+2)1+qpe−(p+q)(tequiv+2)

where the installed base fraction F is bounded by the maximum market share, and m⋅F is the number of adopters. All parameters ($p,\;q,\;t_{equiv},\;m$) are recalibrated at each time step. The historic market size in the training data is introduced on the fly (as it is computed by the model), so that the training set is only “complete” by the last training year (2018). This approach makes the model flexible to input data and does not affect model performance since, in historical years (2014–2018), the model outputs are constrained such that the county-level adoption matches the observed value in the ground truth data. We mitigate issues related to overfitting by placing reasonable bounds on the Bass parameters (Parameter tuning) and determining market size exogenously (Market size).

### Validation

The temporal ground truth dataset described in Data facilitates validation of the model outputs, addressing a gap in the literature. Model calibration uses annual historical adoption data from 2014 through 2018, preserving data from 2019 to 2020 for use in validation. Forecast results for 2019 partly depend on 2020 since they are generated by using the same market conditions with $t_{equiv}$ incremented by one year rather than two as in Equation 6. The validation results presented in the following section assess model accuracy with respect to both number of adopters and system size. We quantitatively validate the model based on mean absolute percentage error (MAPE) across years. This metric can be applied at the county level—where there is relatively more uncertainty in the ground truth data—or any higher level (i.e., national or state). Qualitative validation is performed by observing model response to changing techno-economic conditions (Figure 4) as well as the transition between historical and forecasted years (Figure 3).

While previous models have implemented some combination of (i) calibration and validation, (ii) fine resolution at a large scale, and (iii) forecasting (Table 2), combining all three is a novel contribution of this work. Several extant forecasting models that have been validated operate for single counties with error rates on the order of 10% or better (e.g. (Rai and Robinson, 2015; Zhang et al., 2016)). Recently, Müller and Trutnevyte (2020) offered a larger-scale, national model for Switzerland—which has a population of more than 8.5 million (The World Bank, 2020)—with slightly higher error. Other national-scale efforts have been validated at best spatially (Yu et al., 2018), but not in terms of their forecasting accuracy (Williams et al., 2020). In addition to validating adoption forecast results, we validated the exogenously generated payback periods (Market size) by identifying, state-by-state, discrepancies with a secondary source. Where there was significant disagreement, we updated incorrect or missing incentives. System design decisions made by agents, e.g., the amount of consumption offset by solar generation, were also validated using the same industry source. Originally, agents selected a system size bounded between 0% and 100% of their electricity consumption that maximized their expected net present value. However, data showed that actual adopters have historically sized systems to offset between 50% and 150% their consumption (Fields, Spencer (Energy Sage); Personal Communication [Zoom]. 13 April 2021).

**Table 2:** Summary of past efforts at calibration and validation

Reference	Calibration & Validation	Scale & Resolution	Forecasting interval (predictive error)
(Rai and Robinson, 2015)	Yes	Austin, TX households	Q3 2013–Q4 2014 (5.5%)
(Zhang et al., 2016)	Yes	San Diego County, CA households	05/11–04/13 (∼10%^a^)
(Yu et al., 2018)	Yes	All U.S. census tracts	N/A
(Williams et al., 2020)	No	Germany, Japan, Arizona, California, Massachusetts	N/A^b^
(Müller and Trutnevyte, 2020)	Yes	Switzerland, 143 districts	1 year <12%^c^

a The authors do not provide this figure, but the in the final prediction time period, the average predicted value appears around 125, whereas the true value falls nearer to 139.

b The authors do present predictive results but do not assess their accuracy.

c Estimated based on the best performing model presented by the authors in Figure 9 on page 10. The authors also present 5-year forecasts, but do not give a percentage error.

## Results

Model accuracy is compared across multiple specifications, where calibration is based on historical data from 2014 through 2018 and 2019 and 2020 are reserved for validation (although additional historical adoption data (Calibration) are available for 2010–2013, formulating inputs for the wider dGen model to generate the corresponding exogenous market sizes was beyond the scope of this work). Each resulting specification was used for forecasting PV adoption through 2050 using 2-year time steps. In the following paragraphs, we present the results from various model configurations with an emphasis on evaluating all combinations of the three modifications to the traditional Bass specification. All algorithms are implemented in the Python programming language.

**Figure 2:**
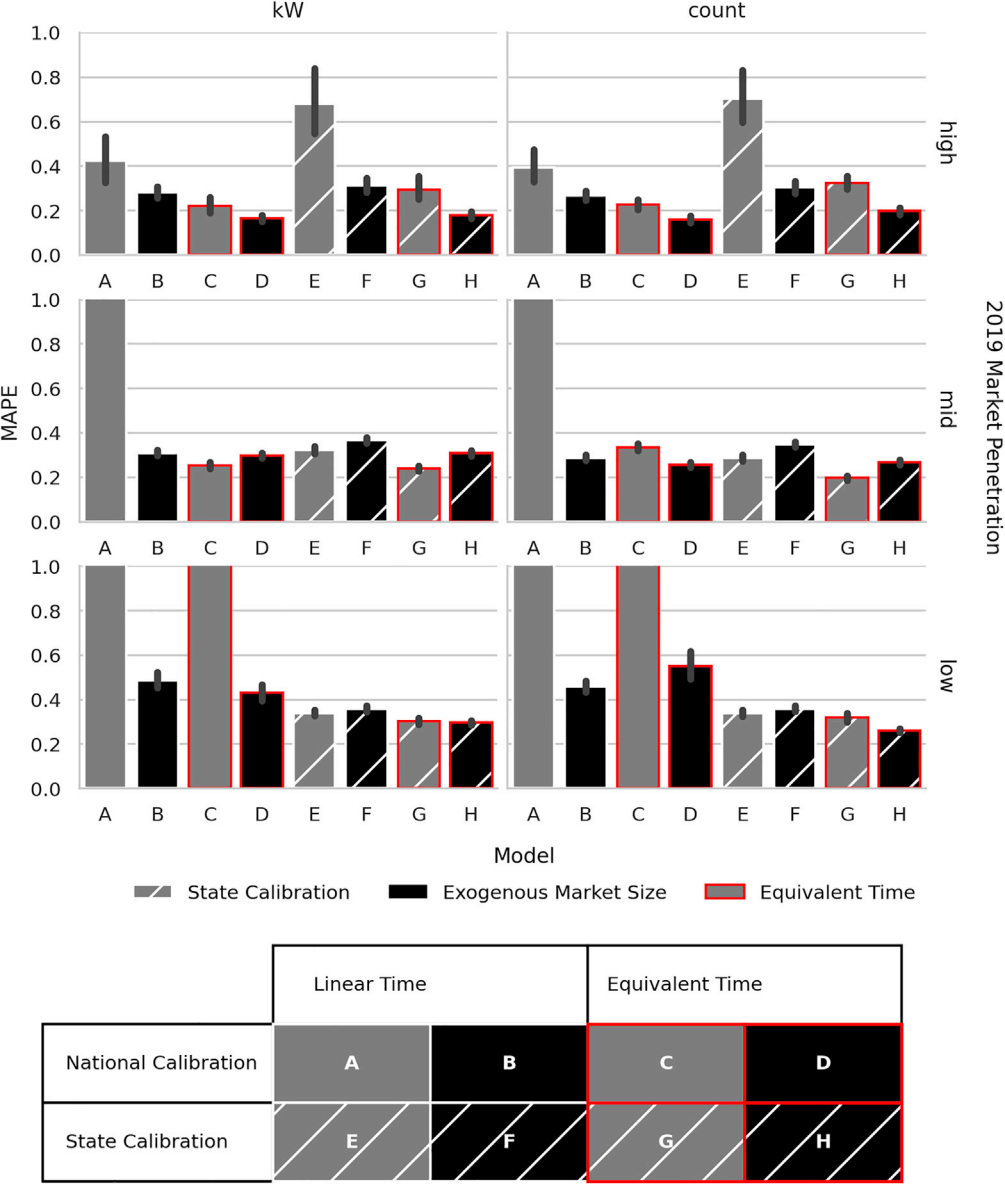
County-level testing error Average county-level error (represented as mean with bootstrapped confidence intervals) for combinations of calibration features faceted by market penetration (low, mid, and high) and unit of prediction (system size in kW and system count). Fill color, outline color, and hashing each represent a single feature—bars differing on a single visual dimension correspond to models differing by a single feature. Models A extends beyond an error of 1.0 at low and mid penetrations; model C does so at low penetrations. Note that three of the top five model configurations use state-level calibration of p and q. Also note the superior performance across market penetration and measure variable of model configuration H. High-penetration states were AZ, CA, CO, CT, DC, DE, MA, MD, NH, NJ, NM, NV, NY, RI, UT, and VT. Medium-penetration states were FL, IA, ID, LA, ME, MN, MO, MT, NC, OR, PA, SC, TX, VA, WA, and WY. Low-penetration states were AL, AR, GA, IL, IN, KS, KY, MI, MS, ND, NE, OH, OK, SD, TN, WI, and WV.

In evaluating each modification to the traditional Bass model, it is useful to compare the possible combinations of traditional and alternative model components. Each configuration combines either (i) state or national calibration of Bass parameters, (ii) exogeneous or calibrated market size, and (iii) linear or equivalent time, Figure 2 (legend); since we do not generate market size at various levels of aggregation this yields eight possible combinations: ***A***-***H***. For example, model ***A*** uses national calibration of Bass parameters, calibrated market size, and linear time while model ***D*** uses national calibration of Bass parameters but exogenous market size and equivalent time. Figure 2 presents average county-level MAPE (2019 and 2020) in system count and capacity for all combinations, faceted by market penetration. Market penetration is measured as the ratio of installed capacity to population at the state-level in 2019, where high, mid, and low represent the top, middle, and bottom thirds of penetration with up to 5, 23, and 148 kW per 1,000 people, respectively. Faceting by penetration highlights how performance can depend on market maturity (e.g., Figure 2 top, model ***C***). While it is conceivable that the COVID-19 pandemic could cause anomalies in solar adoption patterns, solar installations in the United States grew in 2020 and are expected to continue to do so in 2021 due to supportive policies (U.S. Solar Market Insight, 2021) and longer customer-acquisition pipelines, which dampen short-term economic impacts. Combining all the adaptations introduced in the previous section—state-level calibration, exogenous market size, and equivalent time—results in the best-performing model (***H***).

The effect of calibrating Bass parameters at the state—rather than national—level can be understood by looking at each configuration’s average MAPE score across all counties, weighted by population. In the order from best to worst, the best performers are ***H***, ***G***, ***D***, ***F***, ***B***, ***E***, ***C***, and ***A***; ***G*** has marginally lower error in predicting system capacity but higher error in system count than ***H*** and performs worse in high-penetration markets (Figure 2, top). The bottom two models (***A*** and ***C***) use national calibration while three (***H***, ***G***, and ***F***) of the top five use state-level calibration, demonstrating the utility of calibrating Bass parameters at a resolution finer than national scale and supporting the hypothesis that policy, environmental, market, and cultural heterogeneity across the country impact adoption dynamics.

**Figure 3:**
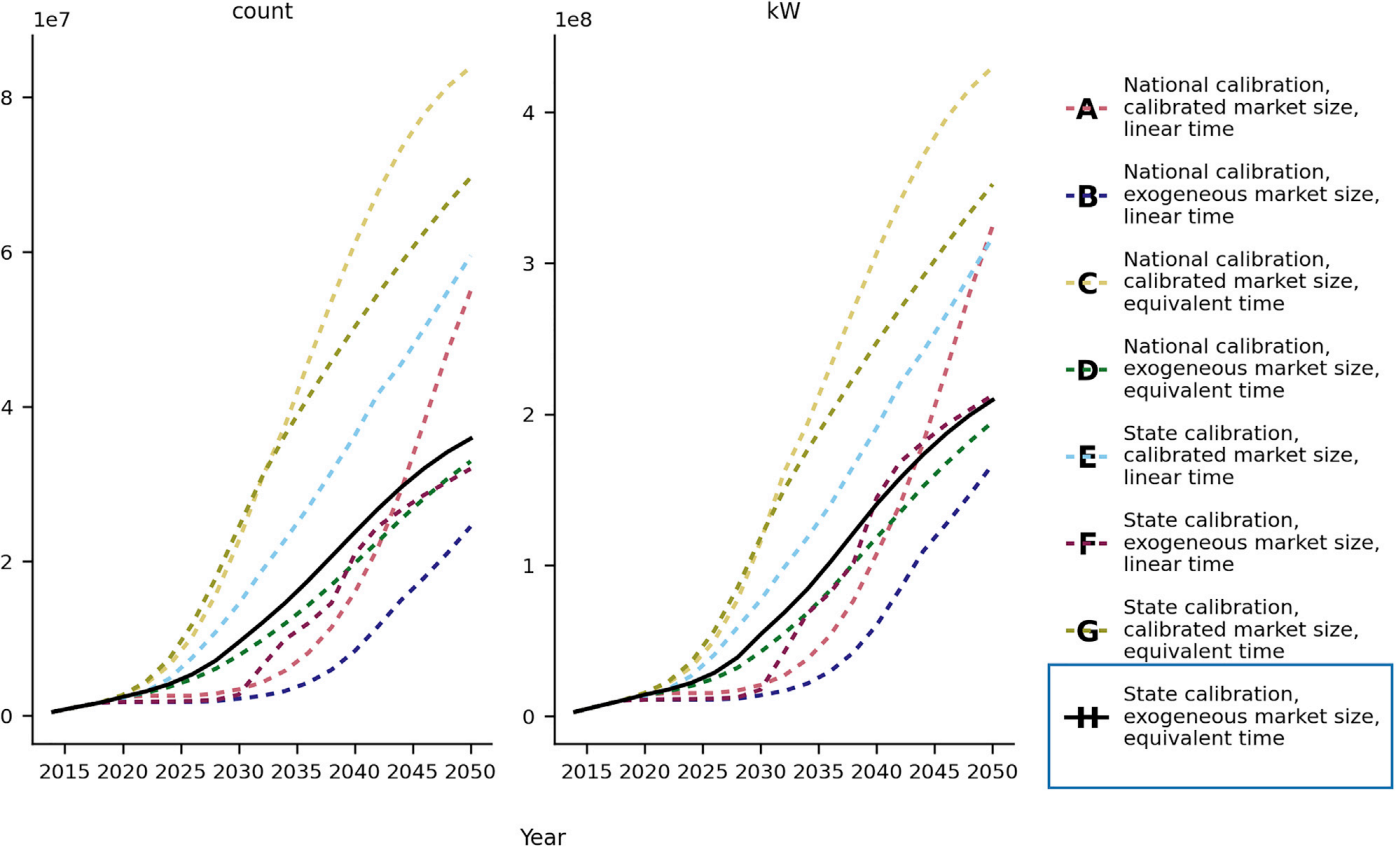
National long-term solar adoption forecasts Forecasts resulting from alternative configurations for number of adopters (count) and cumulative system capacity measured in kilowatts. Note the divergence between different configurations, each using calibrated market sizes (**A**, **C**, **E**, and **G**).

The use of exogenous—in place of calibrated—market sizes results in the most striking difference between configurations. Mirroring the results for Bass parameter calibration, calibrating market size at the national scale (***A*** & ***C***) results in very poor performance in low-penetration markets (Figure 2). Conducting the same calibration at the state level (***E*** & ***G***) gives a better-performing model, especially in low-penetration areas, but still does worse than sibling models with exogenously specified market sizes (***F*** & ***H***). Qualitatively, different configurations, which all calibrate market size, (***A***, ***C***, ***E***, & ***G***) result in highly divergent long-term adoption forecasts; the same is not true of the remaining configurations, which all result in similar forecasts (Figure 3). While this is not a measure of model accuracy, it does provide one indicator of the stability of the model formulation.

The divergence is most likely explained by two factors. The first involves computational artifacts, which we observed when optimizing more than two parameters simultaneously—namely nonconvergence, wherein the algorithm would simply return the midpoint of each parameter’s provided bounds. The second, related, factor is that calibrating—rather than specifying exogenously—market size introduces an additional free parameter during the optimization. This added model flexibility has the effect of increasing test error (Figure 2) due to overfitting to the training data. This is especially relevant with similar numbers of data points (five) and predictors (three: *p*, *q*, & *m*) and demonstrates the utility of exogenously specifying market size when calibrating Bass models on shorter time series. The effect is magnified where diffusion is delayed and the number of data points is effectively reduced. Specifying an exogenous market size offers an additional predictive advantage by capturing future techno-economic conditions (e.g., incentives that are due to expire or block grants that will likely run out of money).

**Figure 4:**
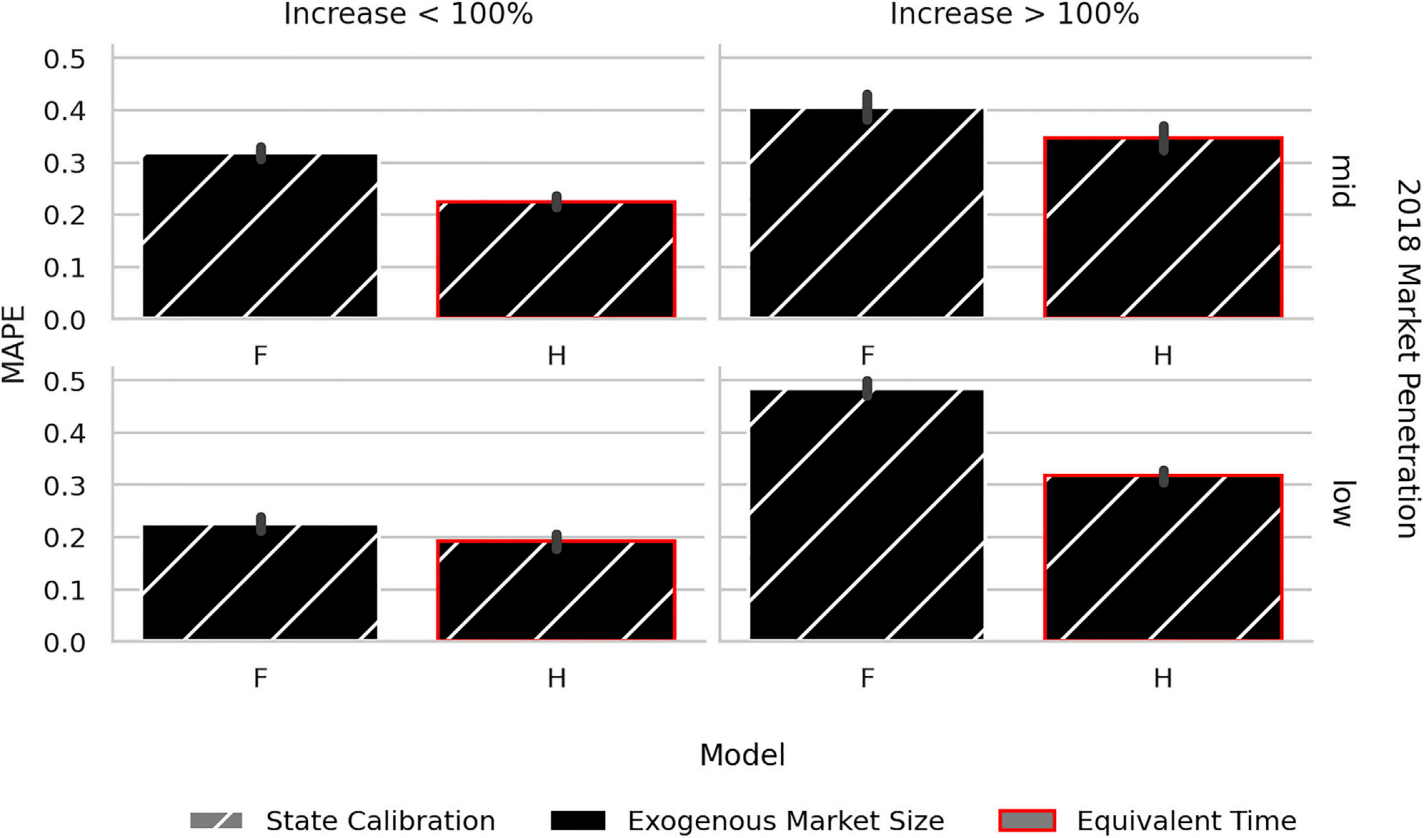
County-level testing error under dynamic market conditions MAPE (represented as mean with bootstrapped confidence intervals) of mid- and low-penetration markets for modeling scenarios **F** (state calibration, exogenous market size, and linear time) and **H** (state calibration, exogenous market size, and equivalent time) faceted by states with dynamic market condition. Reduction in error comes mostly from states where adoption increases drastically (more than the median of 100% over a 2-year period). Low-penetration states that fall into this category are AR, IL, KY, MI, ND, NE, OH, OK, SD, and WV; the medium-penetration states are FL, ID, ME, MT, VA, and WY. No high-penetration states experience more than a 100% increase in adoption over a 2-year period.

The impact of the third modeling choice—equivalent as opposed to linear time—is evident when looking at highly variable adoption patterns. As discussed in Time, the motivation for including this term was to allow flexibility under dynamic market conditions. Such dynamic market conditions occurred, for example, in Illinois where the Adjustable Block Program (part of the Future Energy Jobs Act) began incentivizing small-scale solar at up to $0.0851/kWh in 2019 (Illinois Power Agency, 2020). This led to a significant uptick in adoption in 2019 and 2020. Similar conditions can be found in other states where installed capacity increased by more than 100% from 2018 to 2020 (Figure 4, right). Comparing configuration ***F*** (which uses state-level calibration, exogenous market size, and linear time) to ***H*** (which is the same as ***F*** with equivalent instead of linear time) shows how equivalent time can help with predictive accuracy in these cases. Both variations receive updated (exogenously specified) market size estimates, but the addition of equivalent time makes it possible to “reset” the diffusion curve to reflect the new size of the total addressable market. While ***H*** performs better than ***F*** on average, much of the improvement comes in dynamic markets. Although the performance of ***H*** is improved relative to ***F***, it is still quite poor in low-penetration dynamic markets. Difficulty in capturing dynamic market conditions is compounded by challenges in characterizing lower penetration markets in general where adoption data may be missing for some or all years (because adoption has yet to begin). Importantly, if the change in market conditions is outside the model’s training set (i.e., not included in an exogenous market size specification or historical uptick in adoption) then equivalent time will not have the same benefit. In other words, the equivalent time approach is only useful to the extent that the model is “aware” of changes in market conditions. While challenging and time consuming, maintaining accurate adoption incentives is still necessary for accurate prediction. Additionally, there may be noneconomic factors which increase market size given the same incentive landscape which we do not capture. Dynamically updating the market size based on some combination of the exogenous estimate and historical adoption may provide additional benefits by alleviating both of these shortcomings.

The best-performing model (***H***) combines state-level calibration, exogenous market size, and equivalent time. It is important to recognize that the impact of percent error increases with penetration: a large percentage error in a low-penetration market is likely less impactful than even a small percentage error in a high-penetration area. This configuration has the lowest overall MAPE, but also performs well in high-penetration markets: model ***G***, for example, performs comparably in mid and low-penetration states, but markedly worse in high penetration (Figure 2). For the optimal model, the population-weighted average MAPE for testing (2019 and 2020) and the last year of training (i.e., 2018, when the training set is complete but before the historical constraint is applied) are, respectively, 22% and 15% for system capacity and 19% and 13% for system counts (Table 3). This configuration also exhibits a smooth transition from the historically constrained training years to the forecast years (Figure 3). While our model operates on a larger, national scale, overall county-level error is higher than extant county- or state-level models shown in Table 2. The model performance improves in high-penetration states (at least 28 kW of installed capacity per thousand people) where the average population-weighted county-level MAPE is 13% and 12% for system capacity and count, respectively. In the same set of states, the state-level population weighted MAPE is 8.25% and 9.91% for system capacity and count (3.77% and 5.74% in 2019 and 12.72% and 14.07% in 2020 for system capacity and counts, respectively). These error rates are on par with previous studies in similarly evolved markets. A longer time horizon and irregularities related to the COVID-19 pandemic are likely causes of higher error in 2020 compared with 2019, noticeable in Table 3. The actual 2020 adoption for residential solar was ∼400 MW lower than 2019 forecasts for 2020 (U.S. Solar Market Insight, 2020, U.S. Solar Market Insight, 2021).

**Table 3:** County-level MAPE weighted by population. Breakdown of error rates for optimal model configuration (**H**)

	System Capacity	System Counts
Training (2018)	14.74%	12.91%
Testing — Overall	21.56%	19.24%
2019	18.12%	16.04%
2020	25.01%	22.45%
Testing — High-penetration	12.71%	11.61%
2019	9.01%	8.77%
2020	16.41%	14.46%

Two explanations for the relationship between forecast error and market penetration can be found in the structure of the data. First, lower penetration areas are susceptible to having no adoption in some historical years, reducing the number of useable data points. Second, these areas are likely to have a lower signal-to-noise ratio. Assuming the random variation (noisiness) in yearly adopters is of similar magnitude across geographies, areas with a smaller installed base will, by definition, have proportionally more noise—reducing the accuracy of the fitted model. These results suggest additional challenges with forecasting in low-penetration areas. On the one hand, data are likely to be noisy and possibly missing. On the other, using a less noisy, more complete source (e.g., national adoption as in ***A***, ***B***, ***C***, and ***D***) results in an inferior characterization (Figure 2, bottom).

**Figure 5:**
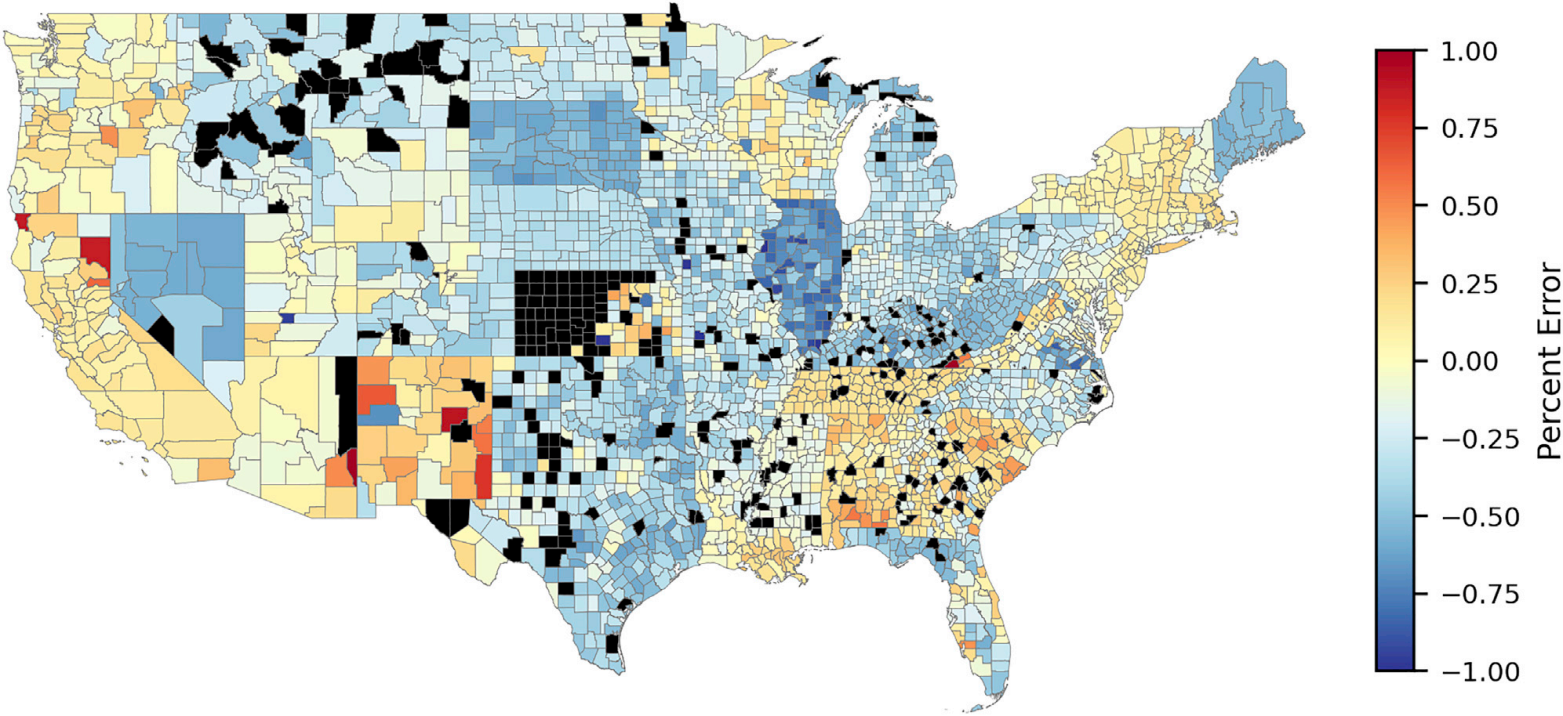
Model H county-level capacity testing error Percent error (PE) in 2019, 2020 adoption forecasting for configuration **H**. The pseudo average is calculated as the mean where the error’s sign is the same for both years, and the larger magnitude otherwise.

A major feature of this model is the ability to forecast and validate PV adoption at county-level resolution for the entire country. Figure 5 presents the nationwide county-level percent error (PE). Counties with no installed solar have an indeterminant PE (they are shaded black). Although they amount to a geographically significant amount of land area, these counties represent only about 0.79% of the national population. As expected, error tends to be lower where market penetration is higher (e.g., the Western Interconnection, Massachusetts, Florida). Other states exhibit very high error, as in the case of Illinois where extremely generous subsidies led to much more adoption than the model predicted or North Dakota, which has very little adoption overall (<200 systems statewide). Forecasting error may be attributed to erroneous imputation in the groundtruth dataset, imperfectly updating market size, and inadequately capturing noneconomic conditions, such as peer effects (Rai et al., 2016; Rai and Robinson, 2013), that drive higher adoption in some areas.

### Value of improved forecasting accuracy

The U.S. power system represents hundreds of billions of dollars in capital investment (EIA, 2021c); thus, even modest improvements to portfolio efficiency have the potential for significant savings. In 2020, the reduction in population-weighted testing MAPE of model ***H*** compared to the baseline (***A***) is more than 170% for system capacity (from 196% to 25%) and 200% for system count (from 226% to 22%). As the forecasts continue to diverge (Figure 3), we expect this error will compound and grow over time. Gagnon et al. provide one way to assign an estimated dollar value to this reduction of error according to the systematic forecast error and increase in distributed solar penetration. Those authors consider a 15-year analysis period in the Western interconnection where, a capacity-expansion plan is developed and implemented based on an erroneous DPV forecast every 5 years. They then compare the present value of capital and operating costs under the erroneous scenario to one with perfect forecasting to estimate the cost of mis-forecasting (Gagnon et al., 2018). Using the 2-year (2020) error as a conservative estimate of the 5-year forecast error and considering the regional change in end-use solar PV market penetration in EIA’s AEO baseline projection (Annual Energy Outlook, 2018), we estimated the penetration-change-dependent cost savings following the results of Gagnon et al. (2018, pp. 26–27). If system planners would otherwise use the baseline model (***A***) and the 2020 difference in error holds, *the optimal configuration would save, over a 15-year period in the Western Interconnection alone, about $167 million as compared to the baseline.* This approach to estimating costs considers only the percent change in penetration and does not consider how financial impacts vary with absolute market penetration.

## Discussion

We present a new method for calibrating and validating a Bass model of technology adoption and implement it in the dGen model (NREL, 2016) to forecast residential solar PV adoption. Alongside the method, we introduce a new ground truth dataset that characterizes residential and commercial historical solar PV adoption in the continental United States. The dataset provides county-level resolution over a 10-year period (2010–2020) at a national scale. Using the ground truth data for validation, we demonstrate the utility of individual components of the new method by implementing them piecewise. Our findings contribute to an understanding of limitations and potential resolutions to the Bass modeling approach.

First, we demonstrate that calibrating Bass parameters separately for different geographies can lead to improved predictive accuracy. This is relevant when working with larger geographies, especially when there is large variance in the degree of market penetration. Second, we find that specifying market size exogenously significantly reduces error. This is particularly applicable when calibrating against shorter time series data where decreased model flexibility. Additionally, using an exogenous market size allows the modeler to include relevant information about the future (during the training period) and further improve prediction. Third, we introduce an “equivalent time” parameter, which adjusts for changes in market conditions by allowing time to advance nonlinearly. We show the effect of including this parameter when market conditions are highly dynamic. Finally, we find model error decreased with market penetration and attribute this to noisy signals and sparse data. Though these improvements are intuitive to some degree, they are also illustrative of the peril of a fitting a simple Bass model to historic data and using it for inference.

Compared to the baseline model, which calibrates Bass parameters and market size at a national scale, our model reduces the error rate from 196% to 25% on average nationwide. This translates to about $167 million in savings over a 15-year period in the Western Interconnection alone. The average testing county-level MAPE for system capacity and counts is 22% and 19% overall and 13% and 12% in states with at least 28 kW of installed capacity per thousand people. These results indicate the importance of considering geographic heterogeneity, data sparsity and noise, and market dynamics when implementing Bass models of technology adoption.

### Limitations of the study

We expect the results presented here to be generalizable to Bass models of other durable goods but a few limitations are worth noting. The accuracy of the model is assessed based on historical ground-truth data, but these data themselves are not free of error (see Data limitations). While the quantitative results surrounding model accuracy will naturally depend on this input data, the qualitative conclusions we draw about modeling approach should not. Also, as we mention above, model performance depends on the quality of exogenous market size estimation—it is expected that inferior and superior estimates will respectively degrade and enhance performance.

## STAR★Methods

### Key resources table

**Table:** 

REAGENT or RESOURCE	SOURCE	IDENTIFIER
**Software and algorithms**		

NREL/dgen: The Distributed Generation Market Demand (dGen) model	GitHub	https://github.com/NREL/dgen
Scikit-learn	PyPI	https://scikit-learn.org/stable/index.html
Scipy	PyPI	https://pypi.org/project/scipy/

### Resource availability

#### Lead contact

Further information and requests for resources and code should be directed to and will be fulfilled by the lead contact, Nicholas Willems (nwillems@utexas.edu).

#### Materials availability

This study did not generate any new materials besides data and code.

### Method details

The Python programming language was used to implement the calibration, validation, and forecasting methodology described in Modeling. Model calibration utilizes several open-source Python packages including Scipy and Scikit-Learn both of which are referenced in the key resources table. The full code and input data are available online.

We derived historical solar adoption data using a combination of the *Tracking the Sun* (TTS) report (Barbose et al., 2019, 2020), the Wood Mackenzie and Solar Energy Industries Association *U.S. Solar Market Insight* (USSMI) (U.S. Solar Market Insight, 2020) report, and the DeepSolar Project (Yu et al., 2018). The combined ground truth dataset describes cumulative and yearly adoption for all counties in the contiguous United States from 2010 through 2020.

To address the underlying incompleteness of the TTS data, we compare the state-level totals to the USSMI dataset. In states where TTS records a higher count, we maintain the granularity therein and treat the data as complete; in states where TTS records a lower count, the additional values from USSMI are imputed across the state’s member counties. Up to 2017, we calculate the (county) difference in system counts between DeepSolar and TTS (2017) and, where positive, use it as a weighting factor for imputation. For the remaining years, we consider all counties with a nonzero number of (previously imputed) installations and impute the additional values from USSMI according to their relative population. See Data for a full discussion.

There are two components to model calibration. First, market size is specified exogenously according to policy and technoeconomic conditions. Second, in order to calibrate the Bass model, agents are grouped by state and combined. For each state, the aggregate installed base fraction F(t) is calculated for each time t based on historical data. Next Bass parameters are fit on these timeseries. Specifically, the coefficients of innovation (*p*) and imitation (*q*) are optimized using a discretized approximation of the differential form of the Bass model:

$$F\left(t+\Delta t\right)\approx F\left(t\right)-\Delta t\left(qF{\left(t\right)}^{2}+\left(p-q\right)F\left(t\right)-p\right)\;$$

where the installed base fraction, F(t), is bounded on either side by 0 and the maximum market share. We restrict the parameters *p* ε [0.0001, 0.002] and *q* ε [0.2, 0.4] to ensure the resulting diffusion patterns are reasonable (these bounds imply that peak adoption occurs [13.7, 38.0] years after the first year of diffusion). Agent adoption is forecast using the resulting market size and the optimized *p* and *q* values. In the validation stage, we compare the forecasted and empirical values. Please see Modeling for a full discussion.

## Data Availability

• Input data are deposited on GitHub and are publicly available at https://github.com/NREL/dgen or https://github.com/nwillems94/dgen/tree/rc5. Tracking the Sun and DeepSolar data are publicly available while the USSMI report is available only for purchase. • All original model code has also been deposited on GitHub and are publicly available at the same links as of the date of publication. • Any additional information may be available from the lead contact upon request.
